# Methionine Sulfoxides on Prion Protein Helix-3 Switch on the α-Fold Destabilization Required for Conversion

**DOI:** 10.1371/journal.pone.0004296

**Published:** 2009-01-27

**Authors:** Giorgio Colombo, Massimiliano Meli, Giulia Morra, Ruth Gabizon, María Gasset

**Affiliations:** 1 Isto di Chimica del Riconoscimento Molecolare, Consiglio Nazionale delle Ricerche, Milano, Italy; 2 Department of Neurology, The Agnes Ginges Center for Human Neurogenetics, Hadassah University Hospital, Jerusalem, Israel; 3 Insto Química-Física Rocasolano, Consejo Superior de Investigaciones Científicas, Madrid, Spain; National Institute on Aging, United States of America

## Abstract

**Background:**

The conversion of the cellular prion protein (PrP^C^) into the infectious form (PrP^Sc^) is the key event in prion induced neurodegenerations. This process is believed to involve a multi-step conformational transition from an α-helical (PrP^C^) form to a β-sheet-rich (PrP^Sc^) state. In addition to the conformational difference, PrP^Sc^ exhibits as covalent signature the sulfoxidation of M213. To investigate whether such modification may play a role in the misfolding process we have studied the impact of methionine oxidation on the dynamics and energetics of the HuPrP(125–229) α-fold.

**Methodology/Principal Findings:**

Using molecular dynamics simulation, essential dynamics, correlated motions and signal propagation analysis, we have found that substitution of the sulfur atom of M213 by a sulfoxide group impacts on the stability of the native state increasing the flexibility of regions preceding the site of the modification and perturbing the network of stabilizing interactions. Together, these changes favor the population of alternative states which maybe essential in the productive pathway of the pathogenic conversion. These changes are also observed when the sulfoxidation is placed at M206 and at both, M206 and M213.

**Conclusions/Significance:**

Our results suggest that the sulfoxidation of Helix-3 methionines might be the switch for triggering the initial α-fold destabilization required for the productive pathogenic conversion.

## Introduction

Prions are the causative agents of fatal transmissible neurodegenerations such as Creutzfeldt-Jakob disease (CJD) in man, bovine spongiform encephalopathy (BSE) in cattle, or scrapie in sheep. Many lines of evidence support PrP^Sc^ as the only essential component of the prion and a protein conformational switch as the basis for its infectivity. PrP^Sc^ differs from its physiological form, PrP^C^, in the C-terminal domain [1]–[3]. This domain adopts a globular and α-helix rich fold in PrP^C^ whereas it is largely aggregated and β-sheet rich in PrP^Sc^ [4], [5]. Once formed, PrP^Sc^ displays its self-perpetuating features by propagating as template its fold to PrP^C^ [6]–[8]. In addition to the conformational difference, PrP^Sc^ also differs from PrP^C^ in the oxidization state of methionine side chains [9], [10]. In particular, the sulfoxidized M213 constitutes a covalent signature for the infectious form [10]. While the conformational change from the α-fold to the β-sheet rich state determines prion amplification properties, the role played by methionine oxidation in the global conversion process remains unknown. Hypothesis on the role played by this modification entail the triggering of conversion, the stabilization of prions, and a marker of reduced clearance, among others [11]–[14].

The oxidation of methionine side chains is a reversible posttranslational modification taking place upon oxidative insults [15], [16]. This reaction transforms the methionine sulfur atom into (*R,S*)sulfoxide groups causing an increase in the side chain hydrophilicity [17]. The resulting sulfoxides can be reduced by the methionine sulfoxide reductase systems [16]. In general, the redox cycle of methionine side chains provides regulatory switches for protein function, structure, assembly and solubility [12], [14], [16], [18]. Previous work has shown that the PrP chain is an excellent substrate for methionine oxidation but the effects of this modification in its conformation and aggregation propensity terms are highly dependent on the experiment setup [10], [19]–[21]. Such variability would be consistent with the high number and differences in location of the methionine residues. In this sense, the HuPrP chain contains nine methionine residues distributed between solvent-exposed loops (M109, M112, M134, M166) and structured regions (M129, M154, M205, M206, M213) anticipating differences in the oxidation susceptibility and in the resulting structural impact [22]. Interestingly, both M206 and M213, both found sulfoxidized in PrP^Sc^ [9], [10], are contained in a chain region that is protected from the solvent and constitutes an amyloid nucleating site [23], [24].

The evaluation of the effect of sulfoxide formation on the structure and stability of the prion protein is a difficult task to approach on experimental grounds. Despite impressive recent improvements there are currently no techniques that can provide atomic insights into the effects of such modification at specific residues. To understand its impact at atomic level computational simulation approaches provide a unique alternative. Using all-atom molecular dynamics (MD) simulations of HuPrP(125–229) and of its oxidation variants, we have found that the oxidation of M213, M206 or the combination of both dramatically changes the global dynamics of the native state, increases the flexibility of specific regions, and alters the network of stabilizing interactions typical for the folded state. The combination of these destabilizing effects increases the probability to populate alternative states which can pave the way to disease-related conversion.

## Results

### Influence of M213 oxidation on structural and flexibility properties of HuPrP(125–229) α-fold

**Figure 1:**
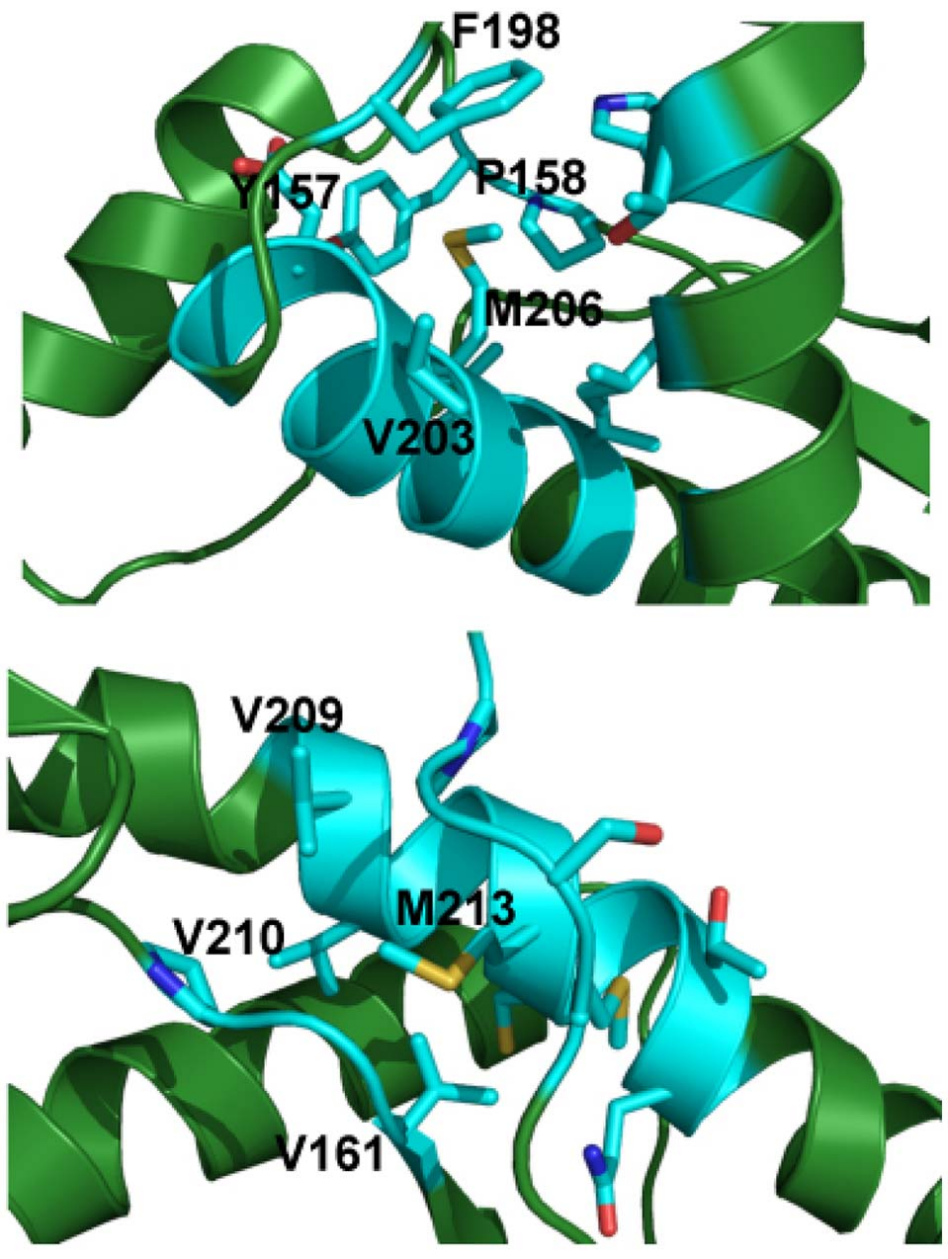
Local environment of M206 and M213 in HuPrP(125–229) α-fold. The residues in close proximity to M206 (upper panel) and M213 (lower panel) are highlighted in a stick representation in light blue. The immediate surrounding of the residues undergoing side chain oxidation is highly hydrophobic.

To investigate the effect of the side chain oxidation of specific methionine residues on the structure and stability of the PrP α-fold we performed a comparative analysis of several time-dependent features obtained from the combination of two different trajectories. Of the different methionines, we start the study using M213 as target of the modification since it was found to be sulfoxidized in the PrP chains of the infectious state [9], [10]. This residue is conserved in all mammalian PrP sequences, forms part of the α-fold mainstay Helix-3 and in this fold the side chain is in contact with hydrophobic residues located mainly on Helix-2 (**Figure 1**) [25].

**Figure 2:**
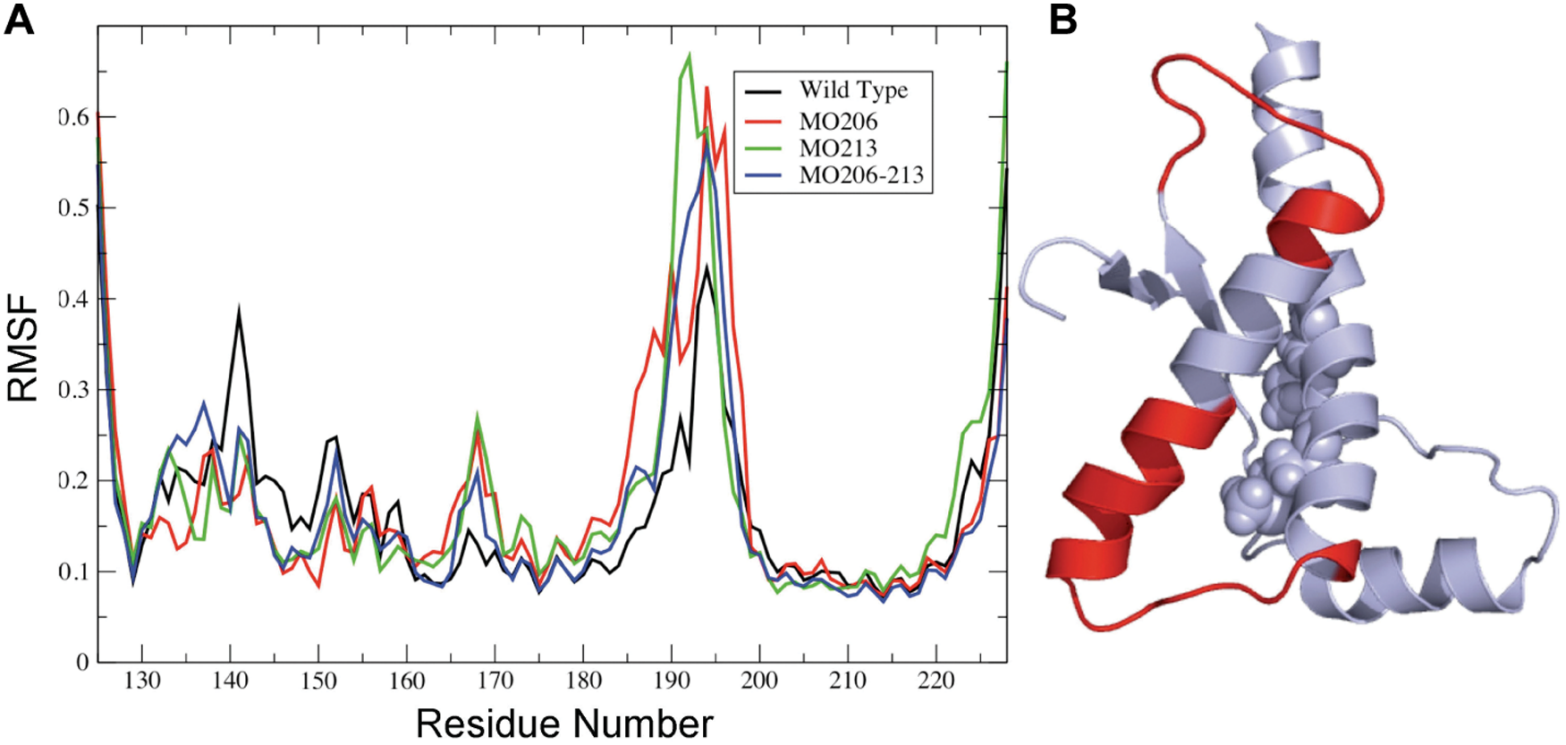
Effect of methionine oxidation on the residue based average flexibility. (A) Variation of the Root Mean Square Fluctuations (RMSF) as a function of the residue number for the different protein forms. The unmodified protein is indicated as wild type, whereas the oxidized forms are indicated as MO together with the location (206, 213 or both). (B) 3D mapping of the regions undergoing flexibility changes. Affected regions are depicted in red, and the Helix-3 conserved methionines are highlighted as van der Waals spheres.

**Figure 3:**
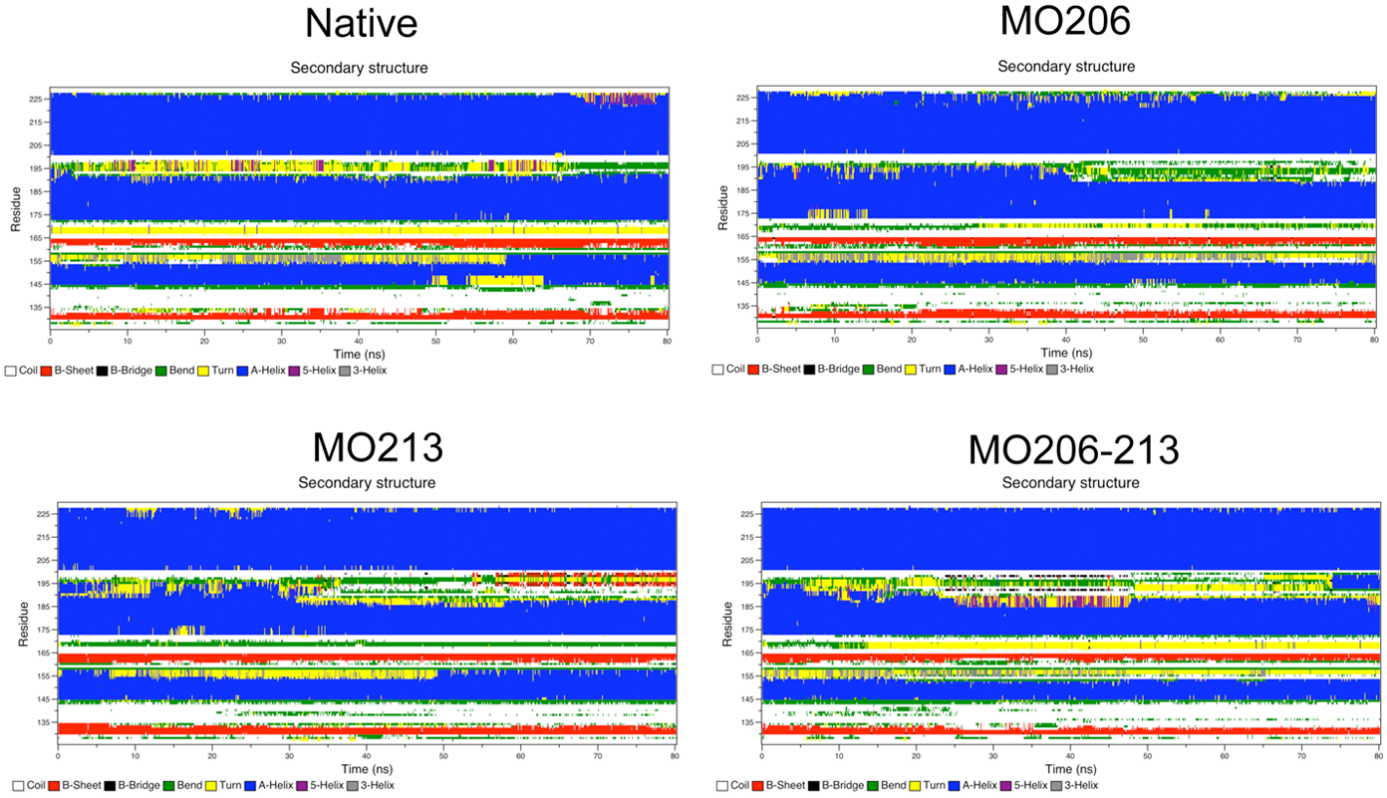
Effect of the side chain oxidation on the time evolution of secondary structure elements. Time evolution of the secondary structure content of unmodified HuPrP(125–229) (Native) and in its oxidized methionine variants (MO206, MO213, MO206–213) calculated according to the DSSP solutions. The color code for the different structure in contained under each panel.

Introduction of a (*S*)-sulfoxide at M213 increases the root mean square fluctuation (RMSF) with respect to that of the unmodified chain in all simulations. The increase in the RMSF, that indicates an enhancement of flexibility, affects mainly the C-terminal part of Helix-2 immediately preceding the site of the introduced modification (**Figure 2A and 2B**). This increase in flexibility is also observed in a limited portion of the N-terminal part of Helix-2 and the loop connecting it to the Strand-2. The analysis of the time-dependent evolution of the secondary structure from the native state shows that the sulfoxidation-induced flexibility increase is paralleled by a partial loss of the native α-helical structure (**Figure 3**). This structural change again affects the C-terminal region of Helix-2, and its unfolding allows the appearance of ordered β-sheet structure involving the sequence stretch covering the C-terminal part of Helix-2 and the loop connecting Helix-2 and Helix-3. In sharp contrast, the simulations of the unmodified chain do not highlight any major conformational rearrangement during the timescale used in the simulation indicating a higher structural stability at both secondary and tertiary levels (**Figure 2** and **Figure 3**).

### M213 oxidation alters global dynamic properties

**Figure 4:**
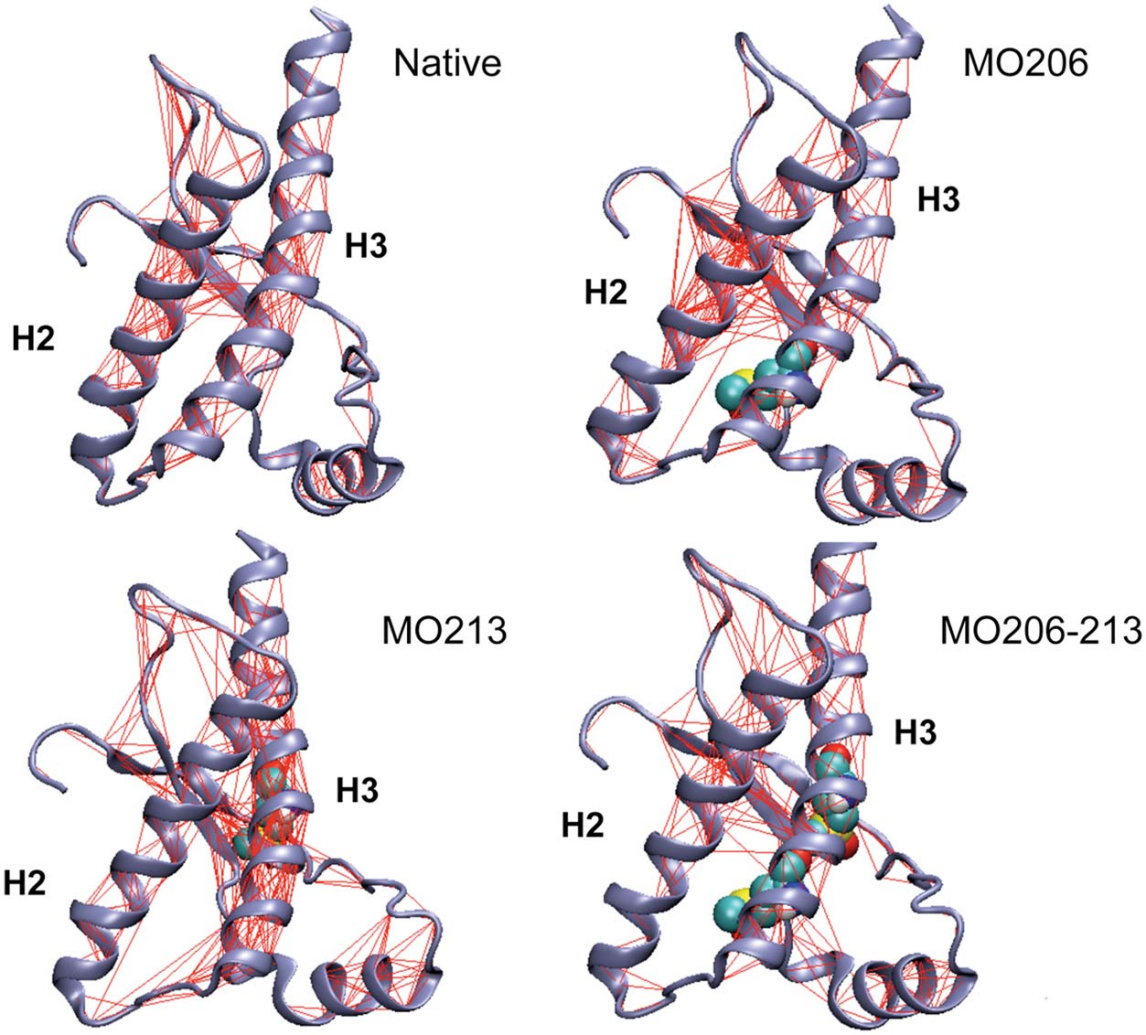
Effect of methionine oxidation on the protein coherent motions. Web-plot of the pairwise covariance matrix accounting for coherent motions in the protein on the 3D structure. The matrix has been computed only considering the Cα atoms. The red lines connect pairs of atoms with a pairwise covariance >0.5. The oxidation sites are highlighted as van der Waals spheres. The picture shows that the presence of oxidized residues strongly influences the parts of the protein undergoing coherent motions.

In addition to the previous changes, the oxidation of M213 could also affect the global dynamic properties. To shed light on this aspect, we carried out Essential Dynamics (ED) analysis on the combined simulation trajectories. From molecular simulations we first calculated the pair wise covariance matrix. This matrix can be used to highlight protein regions that move coherently in a correlated or anti-correlated manner. The covariance matrix can be represented on the 3D structure of the protein by drawing a line between pairs of atoms with a correlation coefficient higher than a given threshold. **Figure 4** displays the projection of the covariance lines on the 3D structure of each of the chains used in the simulations using a threshold of 0.5. This threshold ensures the selection of at least secondary structure elements since their motions are expected to be highly correlated. In the unmodified chain this plot entails mainly the hydrogen bond network typical of secondary structure elements. On the contrary, the plot of the chain containing oxidized M213 shows several extra-secondary structure lines. In particular, additional correlations can be identified between Helix-2 and Helix-3 at the site of the oxidized side chain. Moreover, the decrease in the number of correlation lines detected within Helix-2 indicates the increase of flexibility and the onset of disordered motions in this region of the protein. These findings suggest that M213 oxidation, besides changing the flexibility, impacts on the general dynamical properties of the protein, enhancing the motional correlation between regions playing an important role in the conversion of PrP^C^ into PrP^Sc^ [23], [24], [26].

**Figure 5:**
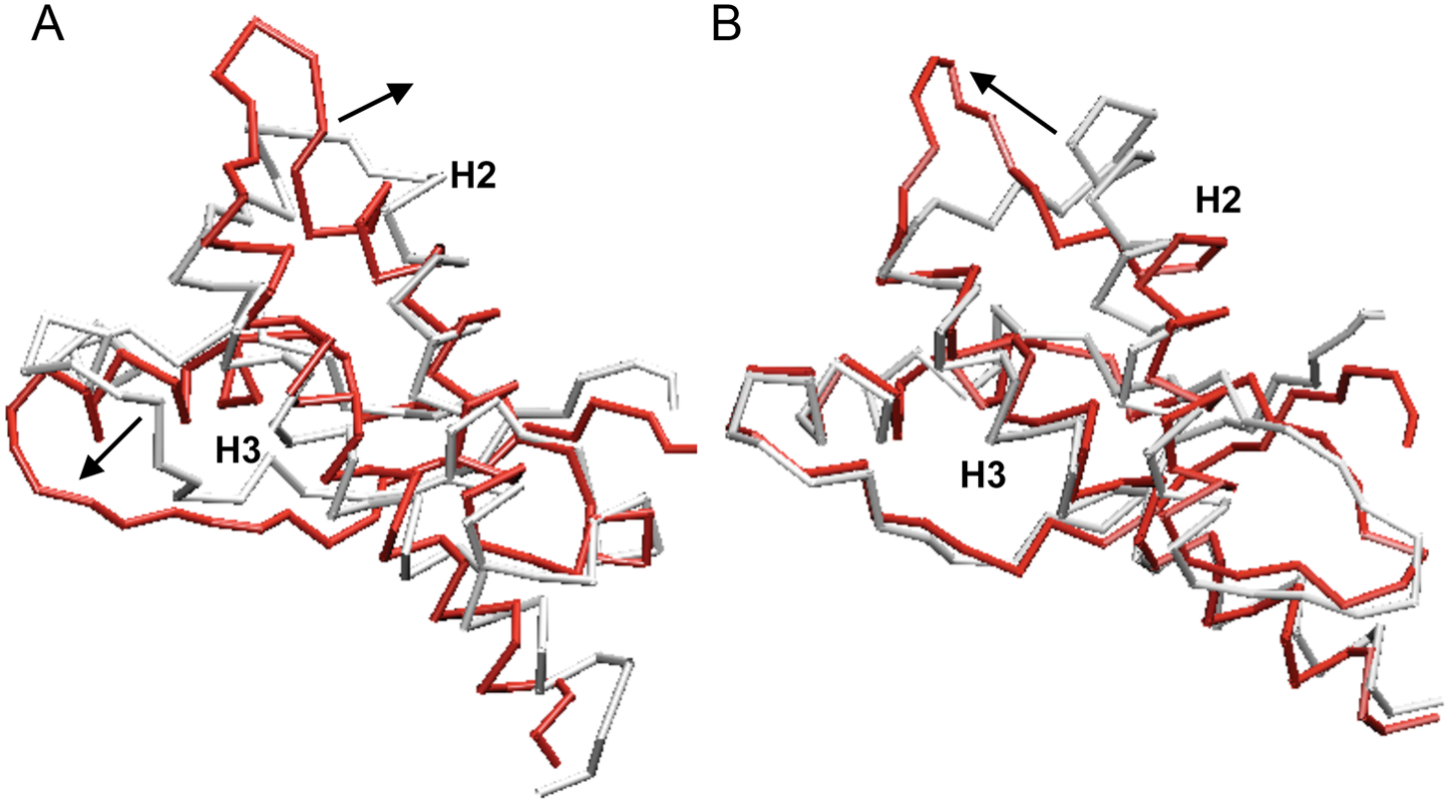
Effect of methionine oxidation on the global dynamic properties of the fold. Projection of the motion along the first eigenvector at 310 K in the (A) unmodified and (B) M213 oxidized proteins. In panel A, the arrows show the directions of the breathing motion. In panel B, the arrows show the direction of the elongation of the loop evolving to a β-sheet structure.

ED analysis reduces the dimensionality of the covariance matrix by diagonalization. This method describes global protein motions that are represented by the matrix eigenvectors and eigenvalues. As opposed to the covariance matrix that highlights regions of coherent atomic motions, ED emphasizes the amplitude and the direction of dominant protein motions. The two analyses do not necessarily coincide since often the timescales of the respective motions can differ even by orders of magnitude. Since the magnitudes of eigenvectors are represented by their eigenvalues, the principal components of the protein global motions can be visualized as a movie by projecting the protein trajectory on the respective eigenvector. For the unmodified HuPrP(125–229) chain the projection of the whole trajectory along the first eigenvector shows that the main modes represent a “breathing” motion of the protein where the N-terminal region, encompassing Helix-1 and part of the original β-sheet, formed by Strand-1 and Strand-2, opens and closes coordinately with the C-terminal part of Helix-2 and the loop connecting Helix-2 and Helix-3 (**Figure 5A**). These correlations are also evident in the fluctuations of the eigenvector components calculated for the first five modes. The main peaks indeed encompass the two regions highlighted above. On the contrary, the main modes of the chains containing the sulfoxidized M213 can be described as a stretching mode of the C-terminal region of Helix-2 and the loop connecting it to Helix-3 (**Figure 5B**). This motion mode is consistent with a local unfolding induced by the perturbation of the protein's energy landscape by the oxidative modification and with the observed variations in the secondary structure content.

### Propagation of M213 oxidation effects

**Figure 6:**
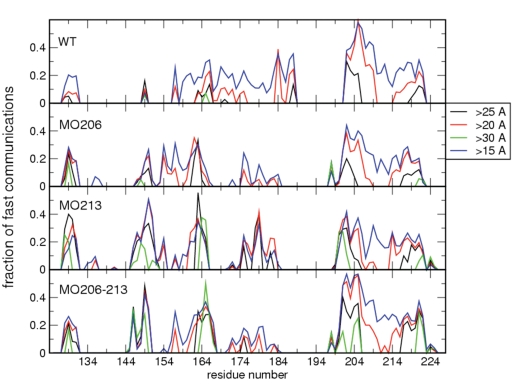
Effect of methionine oxidation on the communication efficiency of all residues at increasing distances. Each bin refers to a residue and shows the fraction of residues of the whole protein that are highly prone to communicate with it (CP<0.025). In each histogram only communications at distances greater than a given threshold (<15 Å, >20 Å, >25 Å, and >30 Å) are considered.

Chemical changes that alter the energy landscape of a protein and permit it to populate different dynamic states within the folded basin, if relevant, reverberate on the communication propensities among pairs of residues [27]. The communication propensity (CP) histograms of the different forms of the protein are shown in **Figure 6**, where each bin represents the fraction of residues that have high communication efficiency with a given residue (CP>0.025) among those with a distance higher than a certain threshold (namely 15, 20, 25 or 30 Å) from that residue. In HuPrP(125–229) the segment comprising Strand-2, Helix-2 and their connecting loop is active in communications with Helix-3 in a range of 15 Å. At increasing distances, the communications are progressively lost and no residue pairs are found to communicate if they are more than 30 Å apart. Sulfoxidation of M213 produces two main changes. First, it switches off all communications involving the loop connecting Strand-2 and Helix-2, as well as those involving the C-terminal part of Helix-2, which becomes more strongly fluctuating. Second, new relevant long distance communications are switched on. The most significant increase in signaling events involves the communications between the extreme edges of Helix-3, namely residues 195–205 with residues 221–225. Moreover, residues 162 to 168, belonging to the loop eventually extending to a β-sheet structure, and residues 201 (N-terminus of Helix-3) and 144 to 150 (the adjacent region of Helix-1) at opposite sides of the protein show an increased communication activity.

Taken together, these results indicate that the sulfoxidation of M213 induces an increase in the signal propagation ability of Helix-3. Thus, the effects of the oxidation-induced perturbations extend well beyond the site of modification and influence the global dynamics of the α-fold increasing its flexibility and the tendency to populate alternative conformations, which in turn may facilitate the productive pathogenic conversion.

### M213 side chain oxidation reorganizes protein energetics

**Figure 7:**
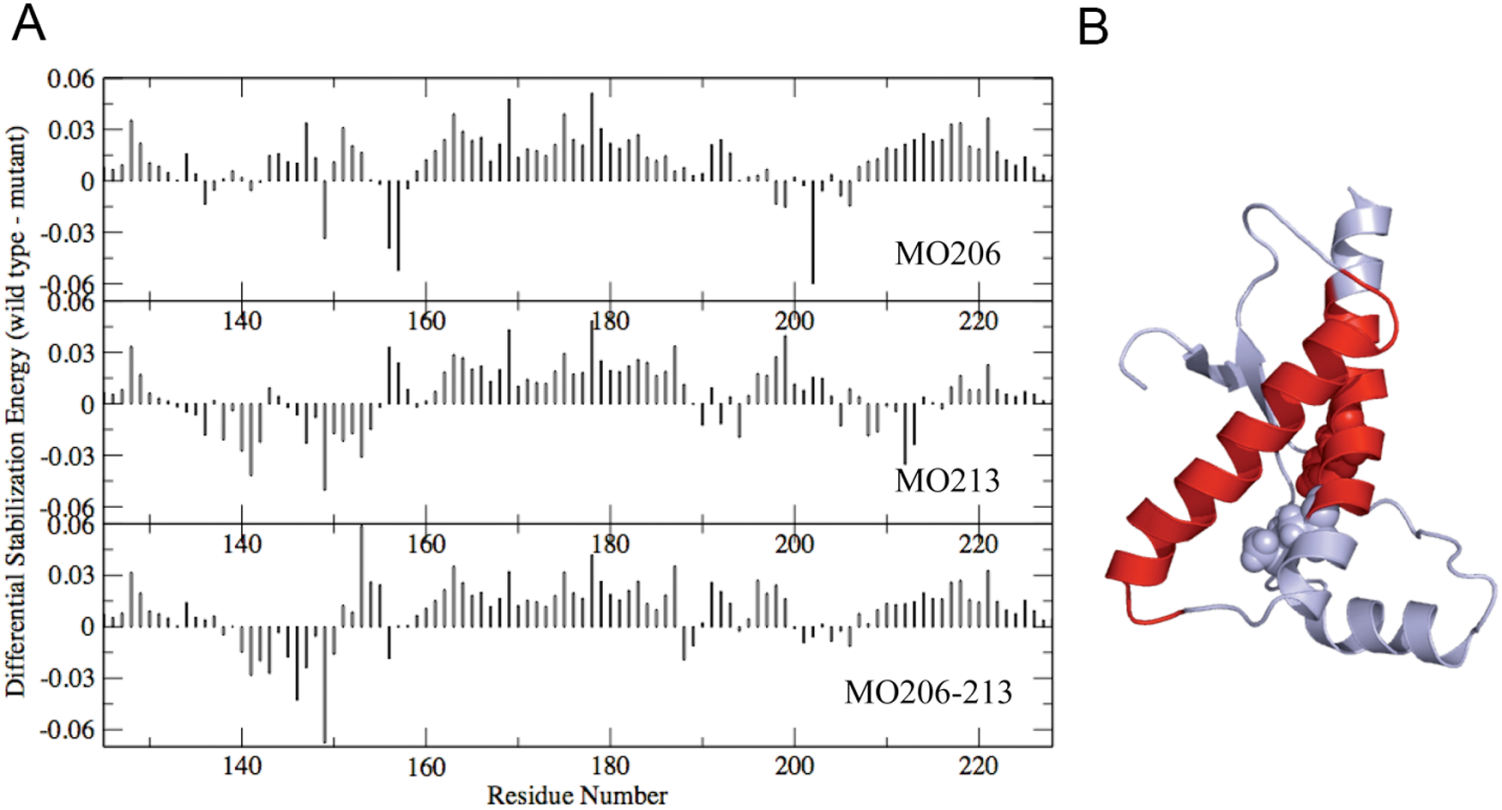
Differential energetics between the unmodified and the oxidized forms. (A) Difference between the respective components of sequence eigenvector (SE) of the unmodified protein minus the SE components of the methionine sulfoxide containing proteins. Positive component indicates that a certain residue is participating in stronger stabilizing interactions in the wild type than in the oxidized form. (B) 3D mapping of the regions undergoing major destabilizations. The native fold is represented in grey, the affected regions in red and the position of the methionine side chains are highlighted as van der Waals spheres.

To gain insight on the effects of M213 oxidation on the protein energetics we analyzed the trajectories from the simulations by an energy decomposition approach aimed at identifying the key residues for the stabilization and folding of the protein [28], [29]. We have previously shown that under native conditions the stabilization energy of HuPrP(125–229) is concentrated in regions of the Helix-1 and Helix-3 [30]. Introduction of a sulfoxide at M213 does not determine a global variation in the appearance of the spectra, with the regions encompassing Helix-1 and Helix-3 concentrating most of the stabilization energy. However, the relative weights of the peaks in the central region of the spectra decrease indicating a parallel reduction in the relative importance of these regions for the protein stabilization (data not shown). To better visualize these changes, the energy decomposition was analyzed as the difference between the eigenvector reporting on the residue-based contribution to stability for pairs of unmodified minus modified proteins (**Figure 7A**). In this graph, positive peaks indicate that the respective position provides a higher stabilizing contribution in the unmodified protein than in the oxidized form. Projection of the resulting variations on the 3D protein structure shows that both Helix-2 and the central part of Helix-3 concentrate most of the stability changes on sulfoxidation of M213 (**Figure 7B**). The specific residues involved in the change of energetics are aromatic amino acids in the surrounding of M213 (**Figure 1**). Oxidation of M213 side chain causes a local change in its polarity, leading to the disruption of the hydrophobic contacts in favor of the formation of hydrogen bonds with R136 and N159.

### The sulfoxidation-induced structural destabilization is a hallmark of conserved Helix-3 methionines

To gain insight into the specificity of the changes observed on oxidation of M213, we inspect the potential effect of such covalent modification at other residues. In addition to M213, HuPrP(125–229) contains six more methionines of which only three (M134, M154 and M206) are conserved in all mammalian sequences. Of these M134 displays high propensity to undergo side chain oxidation appearing as sulfoxide even in the α-fold [10], minimizing the importance of its redox cycle in the conversion process. Similarly, PrP shortened chains lacking the region 141–178 containing M154 behave as PrP^C^ in cells and generate infectious miniprions in transgenic mice [31], [32]. Then, a relevant role for M154 as for any other residue contained in the removed region can be discarded. As opposed to the previous residues, M206 was found oxidized in pioneered mass spectrometry analysis of the PrP chain of highly infectious prion rods and its relevance is unknown [9]. Therefore we performed the analysis of the effect of sulfoxidation at M206 on the structure and stability of the α-fold of HuPrP(125–229).

M206 is located in a structured region (Helix-3) and its side chain in the α-fold is in contact with hydrophobic residues mainly from Helix-2 and the loop connecting Helix-2 and Helix-3. As found for sulfoxidation of M213 but to a lesser extent, the replacement of the sulfur atom by a sulfoxide at M206 increases the structural flexibility of the C-terminal part of Helix-2 and the loop connecting Helix-2 and Helix-3 (**Figure 2**), impacts the general dynamical properties of the protein (**Figure 4**, **Figure 5** and **Figure 6**) and changes the energetics (**Figure 7**). In this case, the local change in its polarity, leads to the disruption of the hydrophobic contacts in favor of the salvation of the oxidized side chain of M206.

Finally, in the absence of experimental evidences supporting a selective modification, we analyzed the effect of the double sulfoxidation replacing the sulfur atoms of M206 and M213 side chains by sulfoxide groups. The obtained results show once more an increased flexibility (**Figure 2**), a generalized and diffuse variation of the global dynamics (**Figure 4**, **Figure 5** and **Figure 6**) and a restructuring of the network of stabilizing energetic interactions (**Figure 7**). Then, these results support that oxidation of any conserved Helix-3 methionines, singly or in combination, switches on the destabilization of the native α-fold.

### High temperature simulation of methionine sulfoxidized variants

**Figure 8:**
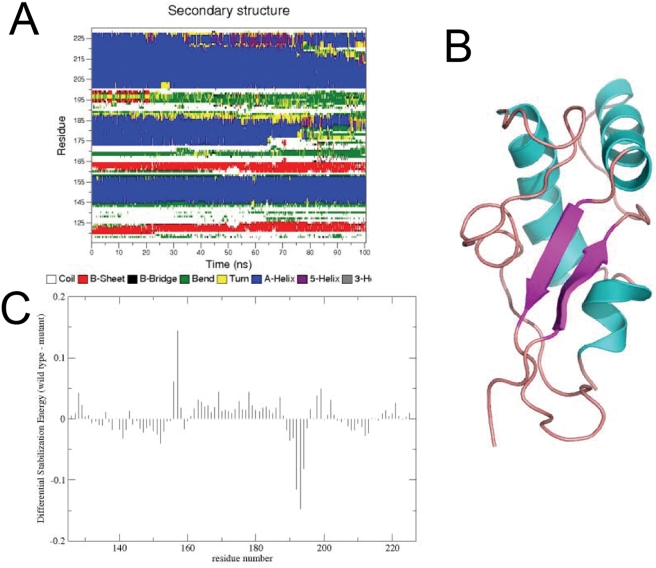
Structural and energetic results of the 380 K simulation of the MO213 containing HuPrP(125–229). (A) Time evolution of the secondary structure content the oxidized methionine variants MO213 calculated according to the DSSP solutions during the simulation at 380 K. The color code for the different structure in contained under each panel. (B) Final structure from the simulation. (C) Difference between the respective components of sequence eigenvector (SE) of the unmodified protein minus the SE components of the MO213 containing protein at high temperature. Positive component indicates that a certain residue is participating in stronger stabilizing interactions in the wild type than in the oxidized form.

Increasing the simulation temperature from 310 K to 380 K should in principle favor structural transitions, possibly leading to partially or completely unfolded conformations more prone to aggregation than the native one. Then a high temperature simulation was run at 380 K for the different sulfoxidized proteins yielding qualitatively analogous results for the three variants (sulfoxidation at M206, M213 and at both sites). Taking the M213 modified chain as example the obtained results are displayed in **Figure 8**. Analysis of the secondary structure evolution shows that complete unfolding cannot be reached even after 80 ns of simulation (**Figure 8A**). However a clear increase in the fraying of Helix-2 and the beginning of the disruption of Helix-3 can be noticed. An increase of disorder in the secondary structure parallels these events. The structure of the final conformation obtained from the simulation highlights an increase of the elongated structure and of the *β*-sheet motifs. Disordered structures appear to flank the preformed sheets and start a pairing process that can eventually lead to an extended β-sheet motif. This can also be reinforced by the parallel disruption of the C-terminal region of Helix-3. The resulting short C-terminal helix defines new contacts with the growing extended region (**Figure 8B**). Finally, the energy decomposition analysis (**Figure 8C**) of the simulation shows the same fundamental properties of the oxidized variants of HuPrP(125–229) discussed for 310 K. However, it is worth noting that the differences in terms of stabilization energy in favor of the unmodified protein in the region of Helix-2 and the central part of Helix-3 are more pronounced with respect to the previous cases. This result suggests that perturbation of the stabilizing interaction involving this region plays indeed an important role in the destabilization of native state, at least in the initial stages of the misfolding process.

## Discussion

The dramatic PrP chain conformational reorganization accompanying the conversion of PrP^C^ into PrP^Sc^ is believed to proceed through a complex pathway involving the obligatory initial destabilization of the PrP^C^ α-fold for its subsequent refolding and assembly into PrP^Sc^. In the absence of a preexisting template that would assist the change, the involvement of a covalent modification, transient or irreversible and affecting either all or a minor subset of molecules, as a trigger was postulated and never discarded [9], [11]. Our previous studies using a combination of protein chemistry and immunological tools have shown that sulfoxidation of M213 constitutes indeed a covalent signature for PrP^Sc^ [10]. This modification was identified in some but not all the PrP chains from the PrP^Sc^ of given specie and it was present in the PrP^Sc^ of all species tested [10]. This non quantitative but state signature characteristic suggested that sulfoxidation of M213 could act as the postulated covalent trigger for the pathological conversion. However, methods such as mass spectrometry of PrP^Sc^ or the immunological characterization of PrP models upon oxidation cannot yet answer the important mechanistic questions of whether such PrP^Sc^ modification constitute more than a marker. The experiments described here, based on a molecular dynamics approach linking covalent modification, conformational properties and possible transition pathways at the atomic level resolution, help to answer such mechanistic questions. It is worth noting that the time scales necessary to probe the full conversion from the α- to the β-fold are clearly inaccessible to all-atom molecular dynamics simulations in explicit solvent at room temperature, and therefore the study has focused on the effect of covalent modifications on the native ensemble properties.

Our results show that the oxidation of either (or both) of the conserved methionines of Helix-3 destabilizes the PrP^C^-like fold favoring the shift towards more flexible states on the energy landscape, characterized by different energetic interaction patterns among the protein's residues. This new dynamic ensemble may be more prone to conformational transitions than the unmodified protein and therefore it may easily populate the productive pathway of the pathogenic conversion. This type of oxidation-induced molecular switch is reminiscent of the ligand-based modulation or allosteric regulation of protein dynamics observed in other systems even in the absence of major conformational changes [33], [34].

The perturbations induced by the oxidative modifications do not have a local effect (i.e. only on the Helix-3 features) but they transmit through the fold to the region preceding the site of modification in the sequence, the loop connecting Strand-2 and Helix-2, the C-terminal region of Helix-2 and part of the loop connecting Helix-2 and Helix-3. This region, either totally or partially, was suggested as one of the sites for conversion based on structural malleability predictions [35]. Moreover, changes in the loop connecting Strand-2 and Helix-2 accompany the opening of the fold into the Strand-1-Helix-1-Strand-2 and Helix-2-Helix-3 halves preceding the onset of PrP self-aggregation [36]. On the other hand, the C-terminal region of Helix-2 and part of the loop connecting Helix-2 and Helix-3 has recently been identified as the binding site for GN8, as small molecules that acting as chemical chaperone stabilize the native PrP^C^ conformation and to prevent its conversion to PrP^Sc^ [26]. Then the oxidation-induced destabilization of both the loop connecting Strand-2 and Helix-2 and the C-terminal region of Helix-2 and part of the loop connecting Helix-2 and Helix-3 looks likely to be an essential step on the productive route of conversion.

We underline that these results have to be considered as qualitative or semi-quantitative, due to the limitation in the time-scales simulated at 310 K. A more complete and thorough sampling of the PrP protein conformational space, possibly simulating reversible folding-unfolding of the protein, must be accessed before fully quantitative estimations of the relative stability of different modified variants can be assessed. These aspects are however out of reach of present all-atom simulations. The results of unfolding simulations must also be considered qualitative as general-purpose force-field parameters have been optimized only for room temperature simulations, and uncertainties may arise with regards to the dynamics of proteins at higher temperature conditions. Importantly, the results of our simulations and analysis show that it is possible to shed light on the effects of covalent modifications on the PrP^C^ native state, and are being used to select point mutations that mimic these effects and to develop MD-based pharmacophore models to identify drugs able to block the pathogenic conversion.

Numerous reports have documented the presence of oxidative damage within the brain in prion diseases suggesting that heightened oxidative stress may play a role in the pathogenesis [37]–[39]. During prion disease, as is the case for aging and other neurodegenerative states, oxidative stress insults might be more efficient resulting in a larger extent of protein methionine oxidation given its scavenger role [13], [15], [40]. Consequently an optimal function of methionine sulfoxide reductases would be required not only for repairing damaged proteins but also for engaging in a cycle of methionine oxidation and reduction that ultimately would results in the scavenging of reactive oxygen species. However, an impairment of methionine sulfoxide reductases surveillance function, as on ageing, would allowed the accumulation of oxidized proteins, as the aberrantly folded PrP^Sc^ [13], [15], [16]. Moreover, since both PrP^Sc^ and oxidized proteins inhibit the proteasome activity, low levels of oxidized PrP may initiate a chain reaction which would ultimate lead to the amplification and conversion required for the prion birth [41], [42].

Overall, our results support the concept that the oxidation of Helix-3 methionines may constitute a crucial effector for triggering the early steps in PrP^Sc^ biogenesis and subsequently underscore the importance of the impairment of the methionine redox cycle at the prion birth.

## Materials and Methods

### Molecular Dynamics

The 3D structure of the HuPrP(125–229) (Pdb entry 1QLZ) was used as a starting point in the simulations [25]. Oxidation variants were generated by replacing the sulfur atom of M206, M213 or both by a (*S*)sulfoxide group. The parameters of the sulfoxide moiety were derived based on previous work [44], [45]. According to the GROMOS philosophy, parameters are in general derived by reproducing the thermodynamic properties of a set of the pure liquids of a series of small molecules containing relevant functional groups. Consistently with the principle of parameter-transferability among analogous functional groups and given the chemical similarities between the -CH_2_-SO-CH_3_ functional group of the oxidized methionine and the CH_3_-SO-CH_3_ molecule present in the GROMOS set of parameters, the charges, van der Waals parameters and the bonded interactions were simply transferred from the CH_3_-SO-CH_3_ to the analogous moiety in the methionine sulfoxide. The validity of the CH_3_-SO-CH_3_ parameters to describe interactions with proteins have also been tested in simulations [45] and confirmed by experimental findings [46]. The topologies and the parameters are available upon request.

For each system, two independent long-time scale all-atom molecular dynamics (MD) simulations in explicit water at 310 K, with simulation times of at least 80 ns were independently run using different sets of initial velocities. Analyses of the structural and dynamical properties were carried out on the total trajectory obtained by the combination of the two simulations for each system. The convergence of each simulation, within the limitations of the simulation timescales, was checked by calculating conformational and energetic properties in time windows of increasing lengths (see **Materials S1**, **Figure S1**, and **Figure S2**). To speed up conformational conversions, one 100 ns control simulation at 380 K, starting from the same initial structure as used for the 310 K simulations, was run for the protein systems. All simulations and the analysis of the trajectories were performed by using the GROMACS software package [47], using the GROMOS96 force field [48] and the SPC water model [49]. The same calculation set up was used for each simulation: to mimic proper solution conditions. The ε-amino groups were considered protonated, while the carboxyl groups were considered to bear a negative charge. The systems were solvated in an octahedral-shaped box large enough to contain 0.9 nm of solvent around each aggregate. Each system was subsequently energy minimized with a steepest descent method for 2000 steps. The calculation of electrostatic forces utilized the PME implementation of the Ewald summation method. The constraining of all bond lengths was performed with the LINCS algorithm [50]. A dielectric permittivity, c = 1, and a time step of 2 fs were used. All atoms were given an initial velocity obtained from a Maxwellian distribution at the desired initial temperature of 310 K. Different initial random seeds were used to start different simulations at a certain temperature. The density of the system was adjusted performing the first equilibration runs at NPT condition by weak coupling to a bath of constant pressure (P_0_ = 1 bar, coupling time τ*_P_* = 0.5 ps) [51]. In all simulations the temperature was maintained close to the intended values by weak coupling to an external temperature bath [51] with a coupling constant of 0.1 ps. The proteins and the rest of the system were coupled separately to the temperature bath. In all cases, the proteins were simulated at 310 K for 80 ns, all simulations were run at NPT conditions.

### Essential Dynamics Analysis

The covariance matrices for the combined trajectories of each of the simulated HuPrP(125–229) forms were built by averaging motions of Cα atoms deviating from the mean structure, with the latter calculated over the trajectory excluding the first 5 ns needed for equilibration. Translational and rotational degrees of freedom are eliminated and the average atomic coordinates, *x_i,ave_*, are calculated along the MD trajectory [52]. The essential directions of correlated motions during dynamics were then calculated by diagonalizing the covariance matrix *C_ij_*.
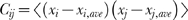



Projecting the MD trajectory onto the main essential direction, corresponding to the largest eigenvector, allows the visualization the extreme structures and the major fluctuations of the correlated motions.

### Analysis of signal propagation

To investigate the modulation of pathways of long-range communication propensities as a function of oxidation we adapted the theoretical approach previously developed for the analysis of signal propagation [53], [54]. The communication propensity (CP) of any pair of residues is defined as the mean-square fluctuation of the inter-residue distance, defining *d_ij_* as distance between the C*α* atoms of residue *i* and residue *j*, respectively:
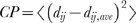



Projection of these quantities on the 3D structures of the protein allows the evaluation of differences in the intra-protein dynamical redistributions of interactions. We set CP = 0.025 as the threshold for discriminating fast communications and used a histogram representation to evaluate the signaling behavior of all amino acids in the protein.

### Analysis of protein energetics variations

The analysis of the variations in the energetics of the oxidized forms with respect to the unmodified form was carried out using the recently developed energy decomposition method [28], [29]. The energy decomposition method is based on the calculation of an interaction matrix *M_ij_*, obtained by averaging the interaction energies between residue pairs, comprising all the non-bonded inter-residue atomic energy components (namely, van der Waals and electrostatic), over a MD trajectory starting from the native conformation. In this calculation, diagonal elements containing self-interactions are neglected. The matrix *M_ij_* can be diagonalized and re-expressed in terms of eigenvalues and eigenvectors, in the form:
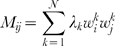
(1)where N is the number of amino acids in the protein, 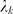 is an eigenvalue, and 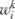 is the i-th component of the associated normalized eigenvector. Eigenvalues are labelled following an increasing order, so that 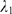 is the most negative. In the following we refer to the first eigenvector as the eigenvector corresponding to the eigenvalue 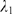. The total non bonded energy *E_nb_* is defined as:
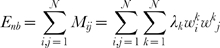
(2)


If the term 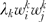 for k>1 is much smaller than 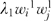, each *M_ij_* can be approximated by the first contribution only:
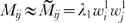
(3)and *E_nb_* can be approximated by *E_nb_^app^*:
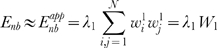
(4)


Analysis of the *N* components of the eigenvector associated with the lowest eigenvalue (eigenvector 1) was shown to single out those residues (hot sites) behaving as strongly interacting and possible stabilizing centers [28], [29]. This procedure allows overcoming the intrinsic complexity of the energy interactions, given by the superposition of many comparable atomic pair interactions. Moreover, it permits the consideration of the modulation of interaction energies due to different structural and dynamical contexts that might not be caught by using more simplified energy models, neglecting motional aspects of protein conformations. The performance of the method and the validity of using the eigenvector associated to the first eigenvalue were previously tested quantitatively on a series of mutants conferring different stabilities to a set of proteins [29]. Using these calculations, the *E_nb_^app^* was shown to account for 61%, 52% and 55% of the stabilization energy for the case of M213 oxidation, M206 oxidation, and double oxidation respectively. In the case of the native protein the approximation was shown to be able to catch 64% of the whole stabilization energy. However, it should be considered that calculation of the native nonbonded energy using the first eigenvalue and associated eigenvector is a simplified approximation of the complex and rough energy landscape of a protein. In this sense, it must also be stressed that while water is explicitly present during the MD simulations, it is not explicitly considered when calculating the average interaction matrix on which the eigenvalue decomposition analysis is applied. The solvent determines the average molecular structures and possible interconversions between different conformations, thus defining the average pairwise interactions characteristic of a certain ensemble of states. Solvation effects are thus considered only in an indirect manner in our energy decomposition analysis. Solvation effects are actually non-pairwise, and considering these factors would have an impact principally on long-range electrostatic interactions. Alleviation of this problem is still under current investigation using implicit salvation approaches.

By applying this method, we derive the vector representation of the amino acid sequence in the native state, showing the contribution of each residue to the stabilization energy. This vector is defined as the Sequence Eigenvector (SE). The energetic effect of methionine oxidation is expressed in terms of the difference between the respective components of SE of the unmodified minus the SE components of the unmodified. The positive components of the resulting difference vector show which residues of the protein are involved in more stabilizing interactions in the unmodified form compared to the oxidized variants, whereas negative components identify residues which are involved in less stabilizing interactions in the unmodified compared to the modified form.

## Supporting Information

Materials S1 (0.02 MB DOC)

Figure S1 Overlay of the residue based RMSF values calculated over increasing time-spans of 20 ns for the combined simulations of methionine sulfoxide containing HuPrP(125–229).
(1.50 MB TIF)

Figure S2 Overlay of the residue based energy components calculated over increasing time-spans of 20 ns for the combined simulations of methionine sulfoxide containing HuPrP(125–229).
(1.20 MB TIF)
