# Supplementary material for: Methionine Sulfoxides on Prion Protein Helix-3 Switch on the α-Fold Destabilization Required for Conversion
Source: PLoS One. 2009 Jan 27;4(1):e4296. doi: 10.1371/journal.pone.0004296 (PMC2628723; doi:10.1371/journal.pone.0004296)
Supplement: Materials S1 — (0.02 MB DOC) [file pone.0004296.s001.doc]

**Supplementary Material**

Structural Flexibility (Figure S1) and components of the first eigenvector (Figure S2) from the energetic decomposition were calculated on windows of different time-lengths from the combined simulations. It is apparent that both the structural and the energetic properties converge in the course of the time spans simulated. Energetic values tend to converge faster than the structural properties, most likely due to the long range nature of the energetic interactions probed.

**Figure S1.** Overlay of the residue based RMSF values calculated over increasing time-spans of 20 ns for the combined simulations of methionine sulfoxide containing HuPrP(125-229).

**Figure S2.** Overlay of the residue based energy components calculated over increasing time-spans of 20 ns for the combined simulations of methionine sulfoxide containing HuPrP(125-229).
