# Supplementary figures and images for: Methionine Sulfoxides on Prion Protein Helix-3 Switch on the α-Fold Destabilization Required for Conversion
Source: PLoS One. 2009 Jan 27;4(1):e4296. doi: 10.1371/journal.pone.0004296 (PMC2628723; doi:10.1371/journal.pone.0004296)

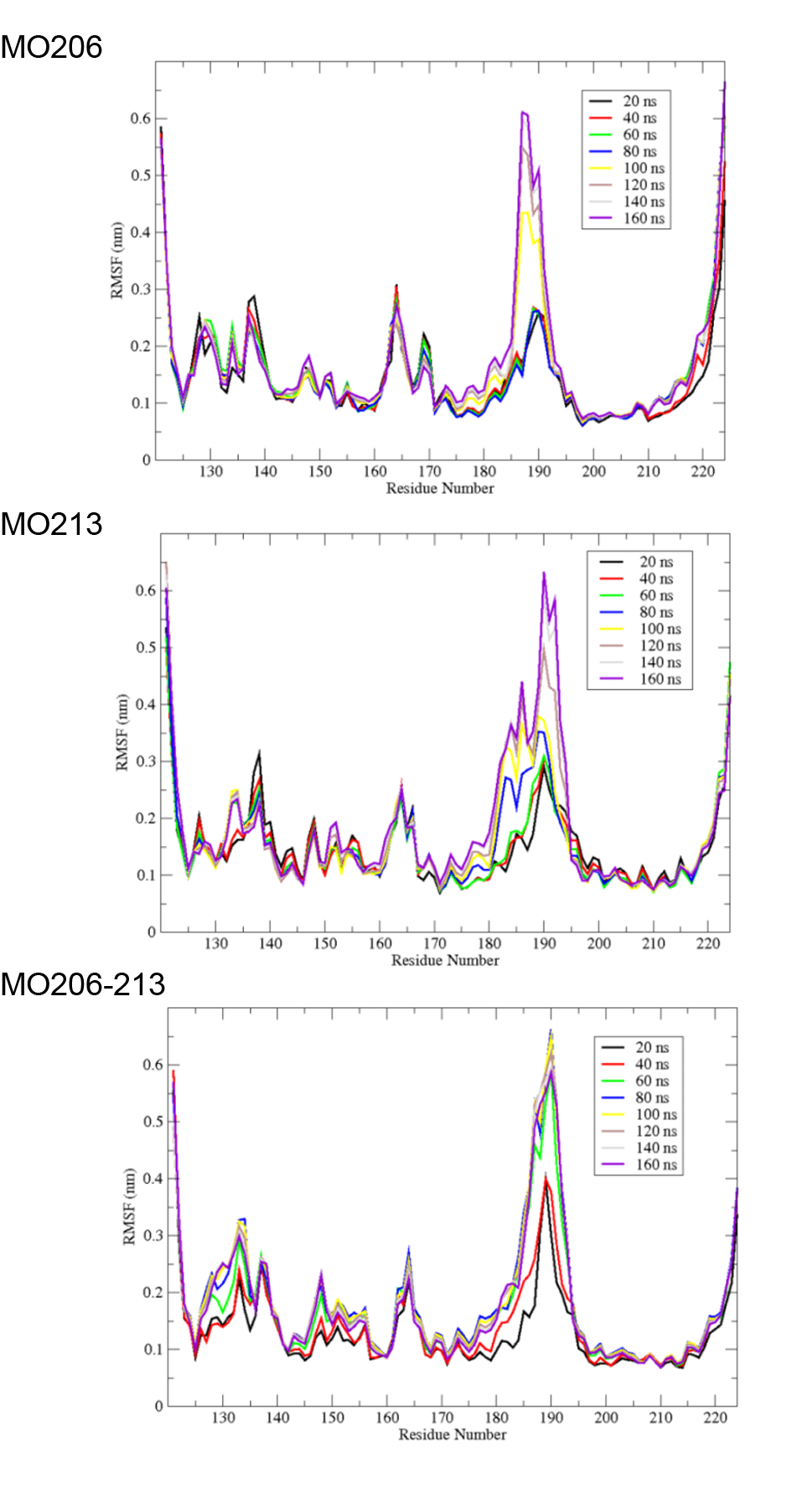

Supplement: Figure S1 — Overlay of the residue based RMSF values calculated over increasing time-spans of 20 ns for the combined simulations of methionine sulfoxide containing HuPrP(125–229). (1.50 MB TIF) [file pone.0004296.s002.tif]

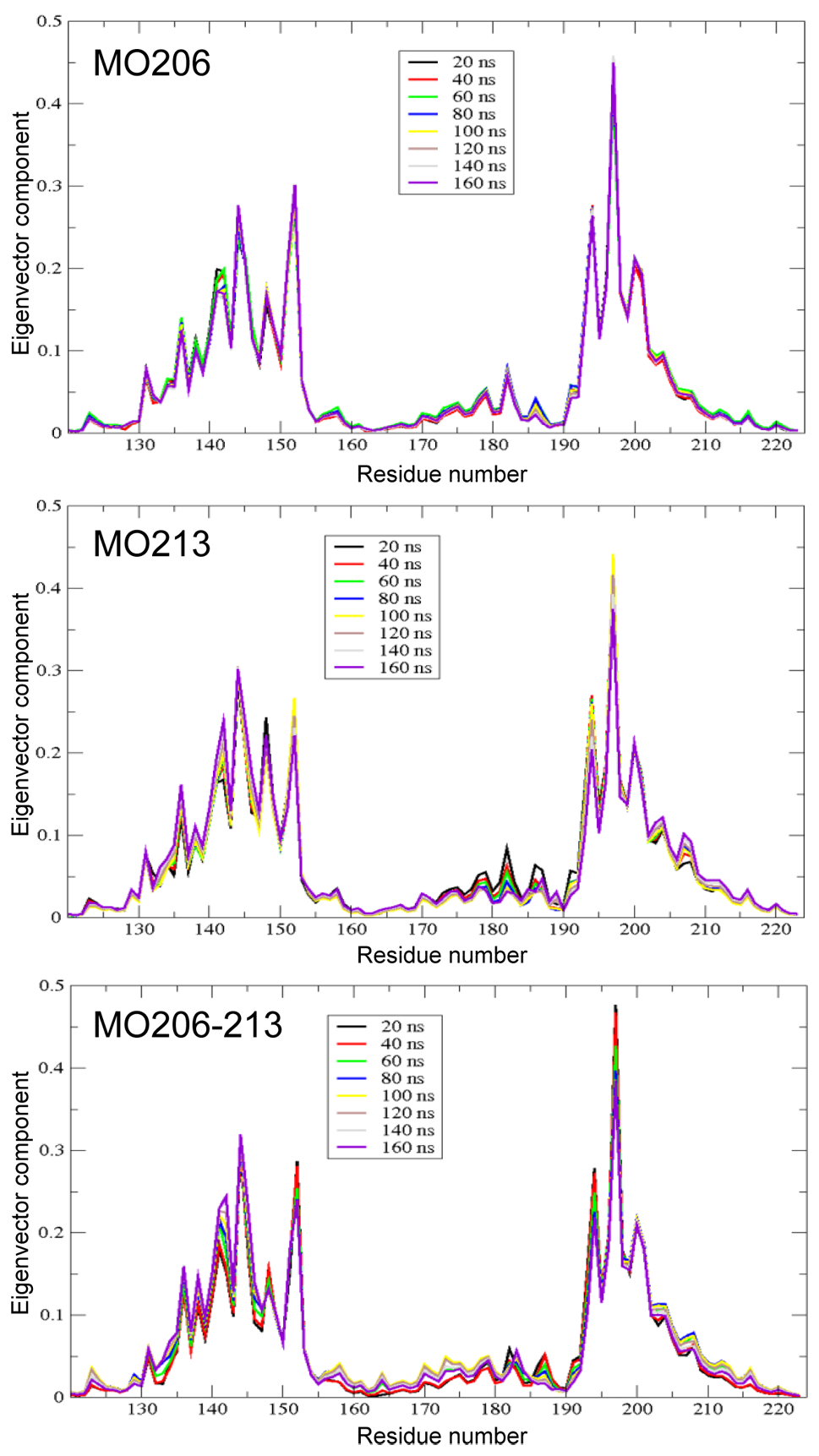

Supplement: Figure S2 — Overlay of the residue based energy components calculated over increasing time-spans of 20 ns for the combined simulations of methionine sulfoxide containing HuPrP(125–229). (1.20 MB TIF) [file pone.0004296.s003.tif]
